# Radiographic results of posterior corrective surgery utilizing 2 different stiffness rods of 6.35mm diameter for adolescent idiopathic scoliosis

**DOI:** 10.1186/1748-7161-10-S1-O70

**Published:** 2015-01-19

**Authors:** Hiromitsu Toyoda, Hidetomi Terai, Akinobu Suzuki, Sho Dohzono, Hiroyuki Yasuda, Koji Tamai, Hiroaki Nakamura

**Affiliations:** 1 Osaka City University Graduate School of Medicine, Japan

## Purpose

The purpose of this study was to analysis the radiographic results of a series of 40 adolescent idiopathic scoliosis (AIS) patients treated by posterior corrective surgery utilizing 2 different stiffness rods of 6.35mm diameter.

## Surgical techniques

There are three major unique points in our technique. 1) Pedicle screws (PS) were inserted when preoperative evaluation revealed that pedicles weren't too narrow for screw placement. Uniplanar or reduction screws were used at the apical concave side for ease in locking rod of concave side. We tended to use high density PS on the convex side. 2) After the PS was positioned, a pre-bend pure-titanium 6.35mm rod was locked to the PSs on the convex side. Subsequently we performed rod derotation and in-situ bending maneuver at the convex side. These can correct deformity in the coronal and sagittal plane. We used pure-titanium 6.35mm rod to prevent fattening while performing rod rotation and for ease in in-situ bending maneuver. 3) After correction of spinal deformity was obtained by convex rod, a pre-bend titanium-alloy 6.35mm rod was locked to the PSs on the convex side to support and maintain the corrected curvature. Vertebral column derotation and compression- distraction maneuver was added after the placement of the second rod.

## Results

This study included 40 patients (3 men and 37 women, the average age was 15.9). According to the surgical classification of AIS by Lenke, the number of patients was as follows: 23 type 1, 2 type 2, 3 type 3, 0 type 4, 10 type 5, and 2 type 6. The average main curve was reduced from 56.8 degrees to 15.2 at the latest follow-up. The mean correction was 73.2%. The average Lumbar compensatory curve was reduced from 34.5 to 11.2 at the latest follow-up. The mean correction was 67.5%. The Thoracic kyphosis was increased from 16.8 to 21.3 at the latest follow-up. The apical vertebral rotation angle was reduced from 22.6 to 17.5 at the latest follow-up.

## Discussion

There were pedicle asymmetry in AIS and generically the diameter of the concave side pedicle is narrower than convex side. Therefore it makes sense to use higher density pedicle screw instrumentation on the convex side and we could demonstrate good correction while performing rod derotation maneuver and in-situ bending maneuver at the convex side. It is a safe and effective method for restoration and maintenance of both coronal and sagittal alignment.

